# *The board is set*, *the pieces are moving*: Modulation of New World arenavirus entry by host proteins

**DOI:** 10.1371/journal.ppat.1009605

**Published:** 2021-06-10

**Authors:** Nicolás Sarute, Susan R. Ross

**Affiliations:** Department of Microbiology and Immunology, University of Illinois at Chicago, College of Medicine, Chicago, Illinois, United States of America; University of Iowa, UNITED STATES

## *Your time will come*: New World arenaviruses are neglected hemorrhagic fever viruses

Arenaviruses are enveloped viruses with 2 ambisense genomic RNA molecules, whose entry into cells is mediated by the viral glycoprotein (GP), generated by proteolytic processing of the precursor GPC into the subunits GP1 (the receptor-binding domain), GP2 (the transmembrane fusion protein), and the stable signal peptide [1]. The family Arenaviridae has been historically represented by a single genus, now named *Mammarenavirus*, members of which are carried by rodent species endemic to the geographic area in which the viruses are found. Recently, the number of genera in this family has expanded to include arenaviruses that infect snakes (*Reptarenavirus* and *Hartmanivirus*) and fish (*Antennavirus*) [2]. The *Mammarenaviruses* are classified into 2 groups: The Tacaribe (New World) serocomplex includes viruses endemic to the Americas, while the Lassa–lymphocytic choriomeningitis serocomplex (Old World) includes viruses indigenous to Africa and Asia, as well as lymphocytic choriomeningitis virus (LCMV), now found worldwide [3]. Members of both complexes are transmitted to humans through direct contact or by inhalation of aerosolized rodent excreta [4]. The New World arenaviruses (NWAs) Junín (JUNV), Machupo (MACV), Sabiá, Chapare, and Guanarito cause hemorrhagic fevers in humans, with about 30% case fatality rate in untreated individuals. A highly effective live-attenuated vaccine for JUNV, the causal agent of Argentinean Hemorrhagic Fever (AHF), has greatly decreased disease incidence in Argentina, although there have been recent discussions about decreasing vaccine production because of low infection rates [5]. Plasma therapy reduces JUNV fatality rates to 1%, but due to the effective vaccination effort, there are now declining numbers of convalescent plasma donors [6]. While only a handful of NWAs are associated with human disease, there are >40 species of rodents harboring >25 NWAs throughout North and South America, and there is a possibility for the emergence of other NWA as zoonoses into humans [3]. Thus, there is still a need for a better understanding of NWA infection and the development of anti-arenavirus therapeutics.

## *Speak the password and the doors will open*: How New World arenaviruses enter cells

**Fig 1:**
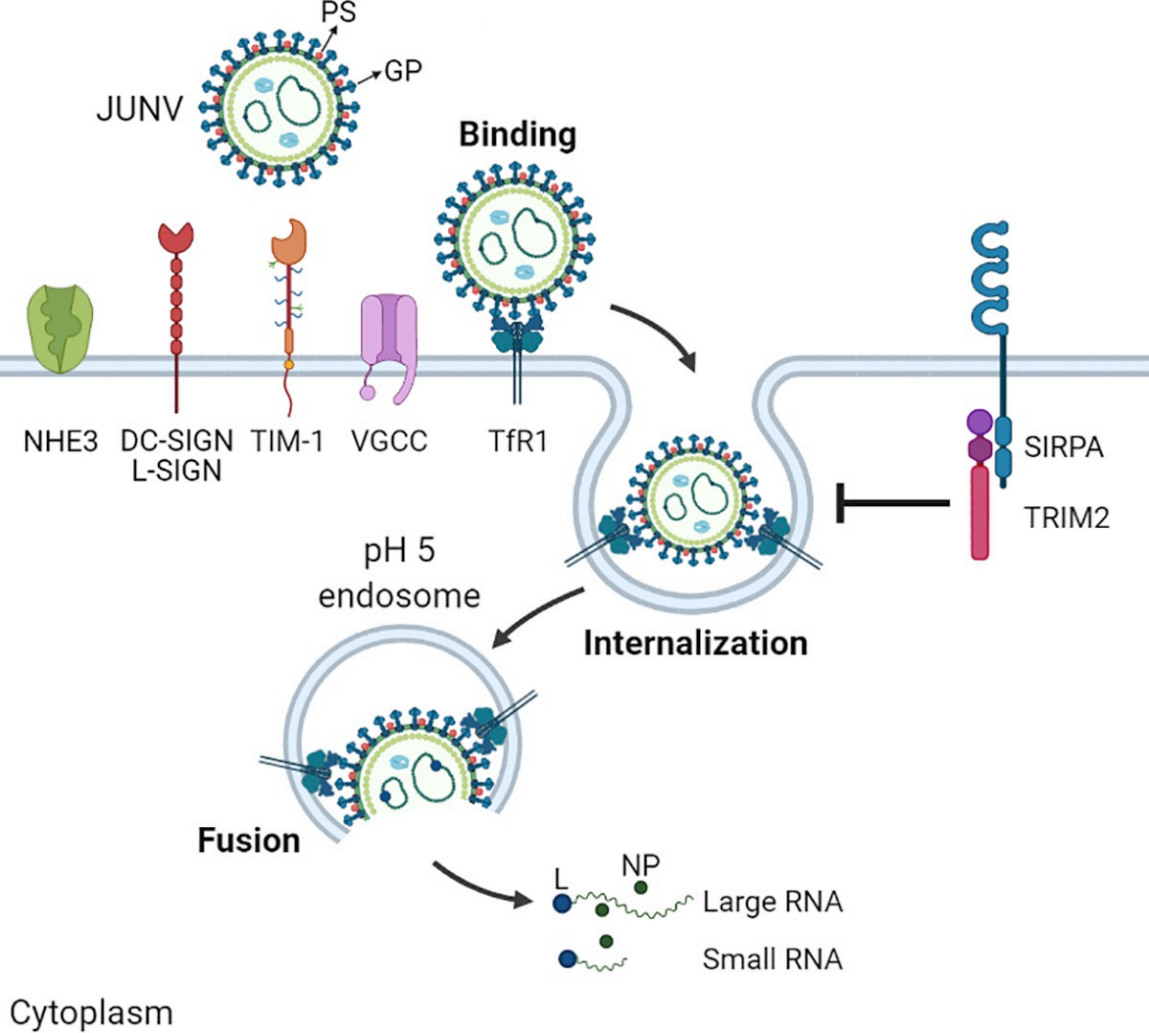
Cellular proteins involved in NWA cellular entry. JUNV uses TfR1 as its main entry receptor on human cells, while it and other NWAs use VGCCs for entry when they cannot use TfR1, such as for infection of mouse cells. NWAs also target additional cell surface attachment factors that enhance binding. NWAs also bind PS receptors, such as TIM-1 due to PS incorporated into viral membranes during budding. Upon binding, virus particles are internalized by endocytosis. The RFs TRIM2 and SIRPA decrease the levels of NWA internalization. Virus-containing endocytic vesicles traffic to acidic endosomes (pH 5) where fusion of the viral and cellular membrane occurs, and the viral genomic RNAs, bound to the L and the NP found in the nucleocapsid, are released into the cytoplasm. Figure created with BioRender (https://app.biorender.com). DC-SIGN, dendritic cell–specific intercellular adhesion molecule-3-grabbing non-integrin; GP, glycoprotein; JUNV, Junín virus; L, polymerase; L-SIGN, liver/lymph node-specific ICAM-3-grabbing non-integrin; NHE3, sodium hydrogen exchanger 3; NP, nucleoprotein; NWA, New World arenavirus; PS, phosphatidylserine; RF, restriction factor; SIRPA, signal regulatory protein alpha; TfR1, transferrin receptor 1; TIM, T cell immunoglobulin and mucin domain; TRIM2, tripartite motif protein 2; VGCC, voltage-gated calcium channel.

Virus entry is a major determinant of cellular tropism, host range, and pathogenesis. Thus, viruses have evolved a variety of entry mechanisms to infect susceptible hosts. The first step of entry involves attachment of the virus particle to the cell surface. NWAs exploit a variety of attachment factors and entry receptors at the cell surface. Some of the exploited attachment factors (proteins, lipids, and glycans) help concentrate virus particles on the surface, while other molecules constitute receptors that actively promote internalization by endocytic processes. Although less specific than receptors, attachment factors can alter the course of infection by increasing the efficiency of virus binding to specific target cells [7]. After receptor binding, arenavirus particles are internalized, and the virus-containing endocytic vesicles traffic to the acidic endosome where entry occurs (Fig 1).

## *And so it begins*: Attachment factors and receptors used by NWAs

NWAs infection is facilitated by cellular attachment factors that enhance binding to host cells. Overexpression of the C-type lectin receptor dendritic cell–specific intercellular adhesion molecule-3-(ICAM-3)grabbing non-integrin (DC-SIGN) and its homologue liver/lymph node-specific ICAM-3-grabbing non-integrin (L-SIGN), which bind carbohydrates in a calcium-dependent manner, enhance JUNV entry in human and mouse cells, owing to direct interactions with the glycosylated viral GP [8]. NWAs can also target phosphatidylserine (PS) receptors to promote their entry. Enveloped viruses acquire PS on the membrane during their replication in host cells, which allows them to disguise themselves as apoptotic bodies and engage PS receptors on target cells [9]. NWAs use the PS receptors T cell immunoglobulin and mucin domain (TIM) 1 and 4 and the TYRO3–AXL–MER (TAM) family tyrosine receptor kinase AXL to enhance their entry [10].

NWAs enter cells through binding specific cell surface receptors that mediate entry. Pathogenic NWAs like JUNV and MACV use transferrin receptor 1 (TfR1) of humans as well as their rodent host species as entry receptors, which is likely why they can be transmitted from rodents into humans [11,12]. However, most NWAs are unable to bind *Mus musculus* TfR1 but infect mice and murine cells by a TfR1-independent mechanism [13]. We showed that L-type voltage-gated calcium channels (VGCC), hetero-multimeric protein complexes that regulate Ca^2+^ influx into cells, are the main entry receptors for NWAs when they cannot use TfR1 on target cells (e.g., JUNV and MACV infection of mouse cells); VGCCs also contribute to infection of human cells by NWAs that efficiently use TfR1 [14,15]. Another protein, sodium hydrogen exchanger 3 (NHE3), a membrane protein involved in receptor-mediated endocytosis, also increases JUNV entry levels in hamster cells, but not in TfR1-expressing human cells [16].

While JUNV and MACV use TIM-1 with moderate efficiency, other NWAs that enter human cells independent of TfR1, such as Tacaribe virus, utilize TIM-1 with high efficiency. It is possible that the interaction of nonpathogenic NWA with VGCCs, their entry receptors on human cells, is not as strong as the NWA GP–TfR1 interactions, thus requiring that these viruses bind additional receptors such as TIM to enhance their entry. This could explain the relatively modest effect of PS receptors on JUNV and MACV entry in human cells [10].

Given the diverse host species and cell types infected by this group of zoonotic viruses, it is highly likely that additional molecules that facilitate NWAs entry in vector species and humans will be identified.

## *You shall not pass*: Cell-intrinsic restriction factors that target NWAs entry

NWA infection is characterized by high levels of pro-inflammatory cytokines and type I interferon production, and it is believed that this host response is in part responsible for disease. The host innate immune system is composed of many molecules that sense infection, including pattern recognition receptors and restriction factors (RFs), which recognize viral proteins and nucleic acids as foreign, thereby triggering these responses as a means of warning surrounding cells to “arm themselves.” These include Toll-like receptors, retinoic acid-inducible gene I (RIG-I)-like receptors and the dsRNA-activated protein kinase R (PKR), among others. Many, but not all these factors, are interferon-inducible genes. NWAs also encode proteins that counteract the host innate immune response; these antiviral proteins are made only after virus protein synthesis commences in the infected cell [17,18].

Although entry is a critical determinant step of viral infection, there are a relatively few RFs that act at this step. We uncovered 2 RFs that act on NWA entry: tripartite motif protein 2 (TRIM2) and signal regulatory protein alpha (SIRPA) [14,19]. TRIM proteins constitute a large family of E3 ubiquitin ligases (>80 members in humans) that have important functions in host defenses against viruses. Many TRIMs are involved in different antiviral responses: regulation of innate immunity, modulation of autophagy-mediated antiviral mechanisms, and targeting of viral components [20]. Using *Trim2* knockout mice, we demonstrated that TRIM2 limits NWA entry in a ubiquitin ligase- and interferon-independent fashion, constituting a unique intrinsic RF within the TRIM family. We also found that SIRPA, a cell surface protein that blocks phagocytosis and is a member of the TRIM2 interactome, works with TRIM2 to decrease arenavirus internalization [19]. *Trim2* knockout in macrophages also alleviated SIRPA-mediated inhibition of phagocytosis of apoptotic cells, thus mechanistically linking virus endocytosis and phagocytosis. Interestingly, we recently found that SIRPA has antiviral activity against a variety of enveloped RNA viruses that traffic to a low pH compartment for entry, including an Old World arenavirus (LCMV), a flavivirus (Zika virus), a filovirus (Ebola virus), and the coronavirus Severe Acute Respiratory Syndrome Coronavirus 2 (SARS-CoV-2), also by limiting their internalization into host cells. Thus, while the TRIM2–SIRPA complex specifically targets NWA entry, SIRPA appears to be a broader modulator of viral entry for viruses requiring acidic compartments. Our current efforts are aimed at determining the mechanism(s) by which TRIM2 and SIRPA impede NWA internalization, as well looking for therapeutics that target the early stages of infection.

## It is a strange fate that we should suffer so much fear and doubt over so small a thing … such a little thing

Since the first outbreaks of AHF caused by JUNV in the late 1950s, there has been considerable progress in controlling the virus through the use of immune sera and the widespread administration of an effective attenuated vaccine. However, there is still a lack of approved antiviral therapies for AHF and other NWA-related diseases, especially critical to the treatment of infection by viruses for which no vaccines are available. As it is very likely that new NWAs outbreaks will occur, we should continue to study this group of neglected viruses, so that we don’t suffer fear and doubt over these “little” pathogens.
